# Microarray dataset of transgenic rice overexpressing *Abp57*

**DOI:** 10.1016/j.dib.2017.07.047

**Published:** 2017-07-23

**Authors:** Lay Wen Tan, Cheng Seng Tan, Zuraida A. Rahman, Hoe-Han Goh, Ismanizan Ismail, Zamri Zainal

**Affiliations:** a Institute of Systems Biology, Universiti Kebangsaan Malaysia, UKM, 43600 Bangi, Selangor, Malaysia; b School of Biosciences and Biotechnology, Faculty of Science and Technology, Universiti Kebangsaan Malaysia, UKM, 43600 Bangi, Selangor, Malaysia; c Biotechnology Research Centre, MARDI Headquarters, P.O. Box 12301, 50774 Serdang, Kuala Lumpur, Malaysia

**Keywords:** Microarray, Abp57, Overexpressing, Rice

## Abstract

The dataset presented in this article describes microarray experiment of *Auxin-binding protein 57*, *Abp57*-overexpressing transgenic rice. The gene expression profiles were generated using Affymetrix GeneChip® Rice (Cn) Gene 1.0 ST Arrays. Total RNA from seedlings tissue of transgenic rice and wildtype, which serve as control were used as starting materials for microarray experiment. Detailed experimental methods and data analysis were described here. The raw and normalized microarray data were deposited into Gene Expression Omnibus (GEO) under accession number GSE99055.

## Specifications Table

**Table:** 

Subject area	Biology
More specific subject area	Gene expression study
Type of data	Table, visualisation figures
How data was acquired	Affymetrix GeneChip® Rice (Cn) Gene 1.0 ST Arrays, Robust Multi-Array Average and statistical analyses
Data format	Raw (CEL.) and normalized (CHP.)
Experimental factors	*Abp57*-overexpressing line vs. wildtype (control)
Experimental features	Total RNA was extracted from whole seedlings of *Abp57*-overexpressing rice and control. Each sample contains three biological replicates and each replicate consists of three seedlings.
Data source location	The National University of Malaysia, 43600 Bangi, Malaysia (3°16′14.63″ N, 101°41′11.32″ E)
Data accessibility	Microarray data are available from Gene Expression Omnibus database with GEO accession number GSE99055

## Value of the data

• Global gene expression analysis of transgenic rice overexpressing an *Auxin-binding Protein 57, Abp57*.
• Provide data basis for identification of genes that might be involved in auxin-regulated activities.
• Guidance for further experiments in analysing biological processes regulated by *Abp57.*

## 1 Data

Total RNA was isolated from *Abp57*-overexpressing rice and control seedlings that were grown under controlled condition for ten days. Affymetrix microarray GeneChip procedures were used to obtain global gene expression profiles of the transgenic rice. The raw and processed data are accessible at NCBI GEO DataSets (doi: https://www.ncbi.nlm.nih.gov/geo/query/acc.cgi?acc=GSE99055).

## 2 Experimental design, materials and methods

### 2.1 Plant material and RNA extraction

T3 seeds of *Abp57*-overexpressing rice obtained through *Agrobacterium*-mediated transformation [1] were used as starting material in this experiment. The control and transgenic rice seeds were surface sterilized and grown on MS media for ten days under controlled condition.

Total RNA was extracted from seedlings of *Abp57*-overexpressing rice and control using TRIzol^®^ reagent (Life Technology, USA) according to manufacturer protocol and then subjected to DNase treatment using Ambion^@^ TURBO^TM^ DNase to remove genomic DNA contamination. Each group of the sample contains three biological replicates and every replicates consisted of at least three seedlings. The quality and quantity of RNA samples was accessed by Nanodrop-1000 spectrophotometer. All samples showed OD_260_/OD_280_ ratios in between 1.830 and 2.004 with concentration ranging from an approximate 126.72 to 290.53 ng/µL. Results from Agilent Bioanalyzer analysis show that all samples have RIN (RNA integrity number) of 8.3–10.0 (Table 1).

**Table 1:** Spectrophotometric reading and RIN of RNA samples.

**Sample**	**Nisbah 260/280**	**Nisbah 260/230**	**Kepekatan (ng/μL)**	**RIN**
Control-1	2.004	1.880	222.17	8.3
Control-2	1.995	1.827	200.68	8.4
Control-3	1.976	1.897	161.13	10
*OxAbp57-1*	1.997	1.859	290.53	9.2
*OxAbp57-2*	1.830	1.628	126.72	8.3
*OxAbp57-3*	1.974	1.981	259.28	10

### 2.2 Microarray data normalization and processing

**Fig. 1:**
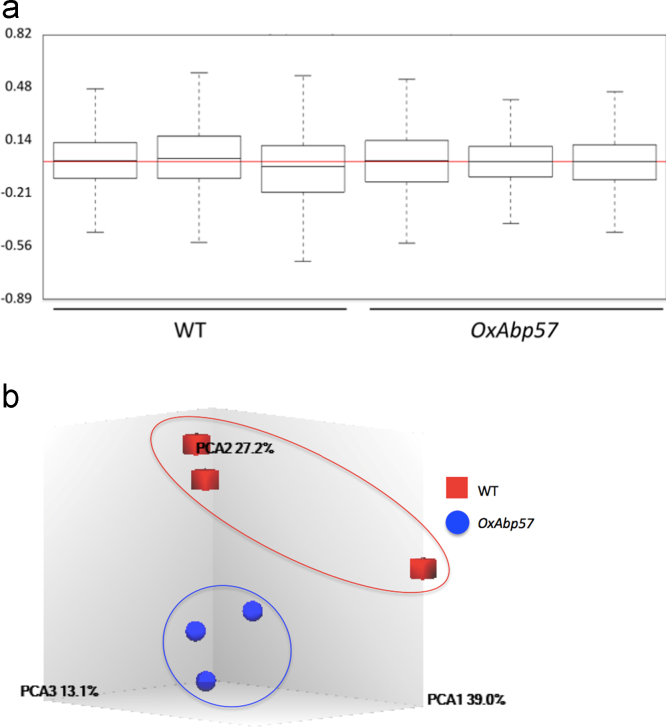
(a) Relative log expression of the samples were found to be at equal level and signal intensity across the samples were comparable. (b) Principal component analysis plot of all six samples. Each dot represents a sample and the positions of the dots are relative to each other. Samples that have similar overall gene expression levels will group together into cluster.

The microarray was conducted with Rice (Cn) Gene 1.0 ST Array (ssp. *indica*) according to manufacturer recommended protocol (Affymetrix, Santa Clara, USA). The CEL files generated were imported into Affymetrix® Expression Console™ Software for normalization using Robust Multiarray Analysis (RMA) method [2] to ensure the comparability of the relative log expression signal (RLE) across all samples (Fig. 1a). Principal component analysis (PCA) showed that the gene expression of *Abp57*-overexpressing rice was distinguishable from the control (Fig. 1b).

**Fig. 2:**
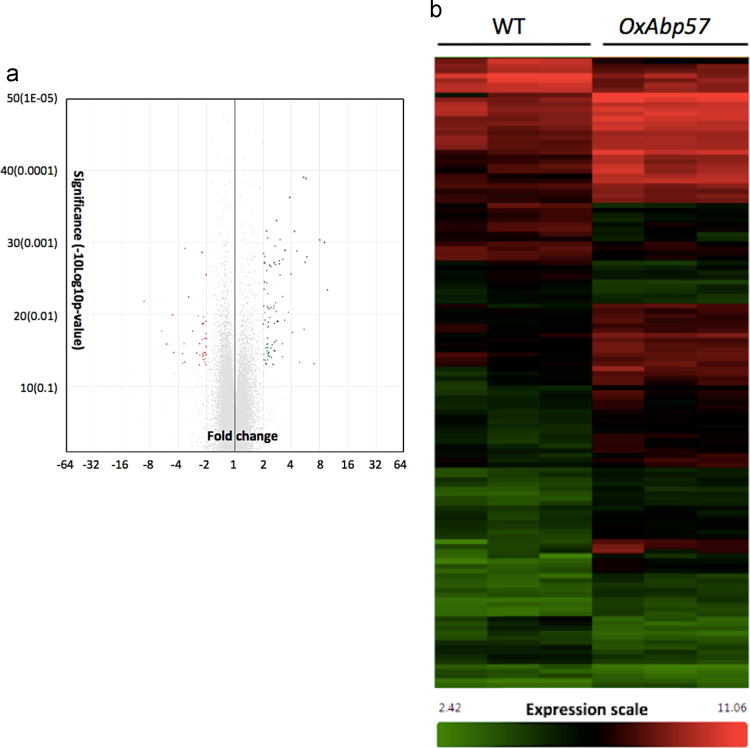
Effects of *Abp57*-overexpression on global gene expression of transgenic rice was profiled using Affymetrix GeneChip® Rice (Cn) Gene 1.0 ST Arrays. (a) Volcano plot showing differentially expressed gene on transgenic rice at absolute fold change >2 and ANOVA *p*-value<0.05. Green dots represent up-regulated DEGs and red dots represent down-regulated DEGs. (b) Heat-map showing expression patterns of 131 DEGs. Red colour columns represent expression level near to 11 and green colour column represent expression level near to two.

The CHP files generated from normalization were subsequently loaded into Transcriptome Analysis Console (TAC) Software for obtaining differential gene expression data using one-way ANOVA (unpaired) and default filter criteria of fold change >|2| with ANOVA *p-value* of less than 0.05. Overexpression of *Abp57* has resulted significant changes in gene expression of transgenic rice as shown in volcano plot and heat-map (Fig. 2). A total of 90 genes were up-regulated and 31 genes were down-regulated in *Abp57*-overexpressing rice compared to control (Supplement 1).
