# Supplementary material for: Microarray dataset of transgenic rice overexpressing Abp57
Source: Data Brief. 2017 Jul 23;14:267–71. doi: 10.1016/j.dib.2017.07.047 (PMC5540701; doi:10.1016/j.dib.2017.07.047)
Supplement: Supplementary file 2 — Supplementary material [file mmc2.docx]

Supplement 1. List of differentially regulated genes between wildtype and *Abp57*-overexpressing rice.

| **Upregulation** | | | | **Downregulation** | | | |
| --- | --- | --- | --- | --- | --- | --- | --- |
| Transcript Cluster ID | Transcript ID | *Fold change* | *ANOVA P-value* | Transcript Cluster ID | Transcript ID | *Fold change* | *ANOVA P-value* |
| 16421073 | LOC_Os11g04020.1; BGIOSGA034557-TA | 9.74 | 0.004522 | 16517406 | LOC_Os04g54210.1; BGIOSGA022083-TA | -2.01 | 0.021473 |
| 16551314 | LOC_Os09g09350.1; BGIOSGA030401-TA | 9.01 | 0.000994 | 16532799 | BGIOSGA024811-TA | -2.02 | 0.027809 |
| 16430617 | BGIOSGA036677-TA | 8 | 0.000918 | 16557958 | BGIOSGA029717-TA | -2.02 | 0.035796 |
| 16480863 | LOC_Os04g28620.1; BGIOSGA016181-TA | 7 | 0.047776 | 16481721 | BGIOSGA016390-TA | -2.03 | 0.002798 |
| 16555722 | LOC_Os09g02270.1; BGIOSGA030237-TA | 5.88 | 0.00159 | 16553251 | LOC_Os09g26900.1; BGIOSGA030839-TA | -2.03 | 0.018303 |
| 16529766 | LOC_Os07g39450.1; BGIOSGA026021-TA | 5.71 | 0.000129 | 16415220 | LOC_Os10g38080.1; BGIOSGA031523-TA | -2.04 | 0.049621 |
| 16471424 | LOC_Os03g18250.1; BGIOSGA010935-TA | 5.6 | 0.001881 | 16420775 | LOC_Os11g01530.1; LOC_Os11g01530.2; BGIOSGA034644-TA | -2.05 | 0.012402 |
| 16460121 | LOC_Os03g19427.1; BGIOSGA012466-TA | 5.47 | 0.016138 | 16528859 | BGIOSGA025830-TA | -2.08 | 0.033581 |
| 16448841 | LOC_Os02g20360.1; BGIOSGA006624-TA | 5.38 | 0.000123 | 16434798 | LOC_Os12g43890.1; BGIOSGA035767-TA | -2.08 | 0.034161 |
| 16481890 | BGIOSGA016429-TA | 4.9 | 0.045984 | 16521448 | BGIOSGA021202-TA | -2.08 | 0.036287 |
| 16527142 | LOC_Os07g15460.1; LOC_Os07g15460.2; BGIOSGA025476-TA | 4.55 | 0.001306 | 16508073 | BGIOSGA017812-TA | -2.08 | 0.041117 |
| 16452251 | LOC_Os02g43410.1; BGIOSGA005930-TA | 4.31 | 0.0007 | 16445433 | LOC_Os02g01750.1; BGIOSGA007288-TA; BGIOSGA007291-TA | -2.08 | 0.045695 |
| 16500266 | LOC_Os05g39540.1; BGIOSGA020031-TA | 4.12 | 0.017697 | 16408013 | LOC_Os10g20450.1; BGIOSGA032737-TA | -2.09 | 0.021412 |
| 16555899 | BGIOSGA030196-TA | 3.95 | 0.002742 | 16384956 | LOC_Os01g22352.1; BGIOSGA003401-TA | -2.13 | 0.033403 |
| 16427113 | BGIOSGA037210-TA | 3.85 | 0.000235 | 16483987 | BGIOSGA016871-TA | -2.15 | 0.01069 |
| 16538204 | LOC_Os07g48770.1; BGIOSGA023691-TA | 3.73 | 0.009196 | 16546713 | BGIOSGA027367-TA | -2.16 | 0.013202 |
| 16388283 | LOC_Os01g46720.1; BGIOSGA004137-TA | 3.43 | 0.001278 | 16565375 | BGIOSGA039874-TA | -2.17 | 0.042061 |
| 16489769 | BGIOSGA015061-TA | 3.39 | 0.011976 | 16459678 | LOC_Os03g17690.2; LOC_Os03g17690.1; BGIOSGA012374-TA | -2.18 | 0.036756 |
| 16566766 | BGIOSGA040495-TA | 3.23 | 0.001708 | 16518806 | LOC_Os06g08240.1; BGIOSGA021788-TA | -2.19 | 0.041869 |
| 16430494 | LOC_Os11g03240.1; BGIOSGA036710-TA | 3.23 | 0.005588 | 16410237 | BGIOSGA033230-TA | -2.21 | 0.034917 |
| 16510092 | LOC_Os06g01250.1; BGIOSGA022122-TA | 3.21 | 0.025581 | 16547937 | BGIOSGA027063-TA | -2.21 | 0.038517 |
| 16531153 | LOC_Os07g46330.1; BGIOSGA026307-TA | 3.2 | 0.038268 | 16428536 | LOC_Os12g36850.1; BGIOSGA037571-TA | -2.23 | 0.013484 |
| 16420860 | LOC_Os11g02440.1; BGIOSGA034619-TA | 3.03 | 0.000916 | 16553174 | LOC_Os09g26620.5; LOC_Os09g26620.2; LOC_Os09g26620.1; LOC_Os09g26620.3; BGIOSGA030826-TA | -2.24 | 0.001365 |
| 16534517 | BGIOSGA024424-TA | 3.02 | 0.002809 | 16524041 | BGIOSGA020640-TA | -2.24 | 0.022051 |
| 16531148 | LOC_Os07g46320.1; BGIOSGA026306-TA | 2.98 | 0.00197 | 16526342 | LOC_Os07g09190.1; BGIOSGA025304-TA | -2.36 | 0.036492 |
| 16473652 | BGIOSGA010463-TA | 2.97 | 0.001794 | 16562983 | LOC_Os08g28670.1; BGIOSGA038814-TA | -2.37 | 0.025256 |
| 16430300 | LOC_Os12g02370.2; LOC_Os12g02370.4; BGIOSGA036763-TA | 2.88 | 0.012501 | 16423679 | LOC_Os11g31540.1; BGIOSGA033924-TA | -2.4 | 0.049497 |
| 16547459 | BGIOSGA027174-TA | 2.84 | 0.012121 | 16383976 | LOC_Os01g15000.1; BGIOSGA003169-TA | -2.46 | 0.010421 |
| 16473657 | BGIOSGA010462-TA | 2.82 | 0.012452 | 16531627 | BGIOSGA026388-TA | -2.56 | 0.034265 |
| 16452246 | LOC_Os02g43370.2; LOC_Os02g43370.1; BGIOSGA005931-TA | 2.77 | 0.000495 | 16478197 | LOC_Os03g61160.5; LOC_Os03g61160.1; LOC_Os03g61160.2; LOC_Os03g61160.4; LOC_Os03g61160.3; BGIOSGA009523-TA | -2.81 | 0.016896 |
| 16393511 | BGIOSGA005178-TA | 2.76 | 0.00904 | 16487929 | BGIOSGA015502-TA | -3.11 | 0.00567 |
| 16406222 | LOC_Os01g72370.3; LOC_Os01g72370.1; LOC_Os01g72370.2; BGIOSGA000097-TA | 2.73 | 0.023122 | 16522249 | LOC_Os06g37300.2; LOC_Os06g37300.1; BGIOSGA021011-TA | -3.39 | 0.045579 |
| 16431533 | BGIOSGA036482-TA | 2.71 | 0.002027 | 16555909 | BGIOSGA030194-TA | -3.4 | 0.001202 |
| 16451134 | LOC_Os02g37654.1; BGIOSGA006150-TA | 2.71 | 0.006832 | 16413998 | BGIOSGA031794-TA | -3.49 | 0.025392 |
| 16461797 | BGIOSGA012818-TA | 2.68 | 0.001123 | 16475144 | LOC_Os03g45960.1; BGIOSGA010107-TA | -3.61 | 0.047309 |
| 16462923 | LOC_Os03g40670.1; BGIOSGA013088-TA | 2.67 | 0.03201 | 16528862 | BGIOSGA025831-TA | -3.62 | 0.034811 |
| 16416069 | LOC_Os10g42180.1; BGIOSGA031361-TA | 2.64 | 0.013267 | 16556121 | LOC_Os09g08130.2; BGIOSGA030146-TA | -4.49 | 0.033352 |
| 16513679 | BGIOSGA022878-TA | 2.62 | 0.001897 | 16450268 | LOC_Os02g32814.1; BGIOSGA006337-TA | -4.63 | 0.010126 |
| 16416436 | BGIOSGA034687-TA | 2.62 | 0.031399 | 16413996 | BGIOSGA031795-TA | -5.35 | 0.025654 |
| 16398547 | LOC_Os01g28030.1; BGIOSGA001608-TA | 2.61 | 0.007496 | 16428530 | LOC_Os12g36830.1; BGIOSGA037569-TA | -6.05 | 0.016867 |
| 16430647 | LOC_Os12g03899.2; LOC_Os12g03899.3; BGIOSGA036673-TA | 2.61 | 0.024162 | 16382979 | LOC_Os01g10400.2; LOC_Os01g10400.1; BGIOSGA002954-TA | -9.27 | 0.006517 |
| 16421186 | LOC_Os11g04320.1; BGIOSGA034535-TA | 2.6 | 0.049977 |  |  |  |  |
| 16471695 | LOC_Os03g19420.2; BGIOSGA010884-TA | 2.58 | 0.008073 |  |  |  |  |
| 16432337 | LOC_Os04g11970.1; BGIOSGA036321-TA | 2.52 | 0.048372 |  |  |  |  |
| 16462343 | LOC_Os03g31730.1; BGIOSGA012934-TA | 2.49 | 0.026251 |  |  |  |  |
| 16537109 | BGIOSGA023888-TA | 2.45 | 0.039178 |  |  |  |  |
| 16497327 | BGIOSGA019406-TA | 2.43 | 0.007866 |  |  |  |  |
| 16547462 | LOC_Os08g24300.1; BGIOSGA027173-TA | 2.42 | 0.002166 |  |  |  |  |
| 16542470 | LOC_Os08g34210.1 | 2.42 | 0.029031 |  |  |  |  |
| 16500263 | BGIOSGA020030-TA | 2.36 | 0.011068 |  |  |  |  |
| 16396421 | LOC_Os01g12400.2; BGIOSGA002098-TA | 2.35 | 0.008302 |  |  |  |  |
| 16430482 | BGIOSGA036713-TA | 2.34 | 0.002067 |  |  |  |  |
| 16443098 | LOC_Os02g49870.2; LOC_Os02g49870.1; BGIOSGA008985-TA | 2.33 | 0.037879 |  |  |  |  |
| 16461282 | BGIOSGA012703-TA | 2.3 | 0.033367 |  |  |  |  |
| 16504665 | LOC_Os05g12400.1; BGIOSGA018580-TA | 2.28 | 0.012701 |  |  |  |  |
| 16416636 | BGIOSGA034739-TA | 2.27 | 0.014534 |  |  |  |  |
| 16561373 | BGIOSGA038186-TA | 2.27 | 0.033912 |  |  |  |  |
| 16428719 | LOC_Os12g37650.1; BGIOSGA037602-TA | 2.26 | 0.008136 |  |  |  |  |
| 16461537 | BGIOSGA012766-TA | 2.25 | 0.028845 |  |  |  |  |
| 16494119 | LOC_Os04g55180.1; LOC_Os04g55180.2; BGIOSGA014235-TA | 2.25 | 0.03484 |  |  |  |  |
| 16490056 | LOC_Os04g37580.1; BGIOSGA015000-TA | 2.25 | 0.037015 |  |  |  |  |
| 16430677 | LOC_Os12g04120.1; BGIOSGA036668-TA | 2.25 | 0.042202 |  |  |  |  |
| 16552837 | BGIOSGA030746-TA | 2.24 | 0.005119 |  |  |  |  |
| 16547856 | LOC_Os08g27840.2; BGIOSGA027081-TA | 2.24 | 0.015093 |  |  |  |  |
| 16425836 | LOC_Os12g03270.1; BGIOSGA036926-TA | 2.24 | 0.02539 |  |  |  |  |
| 16519766 | LOC_Os06g12560.1; BGIOSGA021605-TA | 2.23 | 0.000869 |  |  |  |  |
| 16487845 | BGIOSGA015525-TA | 2.23 | 0.007722 |  |  |  |  |
| 16393758 | LOC_Os01g73024.1; BGIOSGA005224-TA | 2.23 | 0.021114 |  |  |  |  |
| 16459649 | LOC_Os03g17600.1; BGIOSGA012367-TA | 2.21 | 0.032037 |  |  |  |  |
| 16452178 | LOC_Os11g43860.2; LOC_Os11g43860.1; BGIOSGA005943-TA | 2.2 | 0.0258 |  |  |  |  |
| 16484130 | LOC_Os04g46780.1; LOC_Os04g46780.2; BGIOSGA016897-TA | 2.19 | 0.028513 |  |  |  |  |
| 16383160 | BGIOSGA002993-TA | 2.18 | 0.002432 |  |  |  |  |
| 16461474 | BGIOSGA012750-TA | 2.18 | 0.003605 |  |  |  |  |
| 16490877 | LOC_Os04g41340.1; BGIOSGA014826-TA | 2.17 | 0.000693 |  |  |  |  |
| 16468769 | LOC_Os03g06090.1; BGIOSGA011423-TA | 2.16 | 0.049105 |  |  |  |  |
| 16421102 | LOC_Os11g04104.1; LOC_Os11g04104.2 | 2.13 | 0.047274 |  |  |  |  |
| 16517940 | LOC_Os06g04510.2; LOC_Os06g04510.1; BGIOSGA021980-TA | 2.11 | 0.029137 |  |  |  |  |
| 16415989 | LOC_Os10g41780.3; LOC_Os10g41780.1; BGIOSGA031378-TA | 2.1 | 0.04283 |  |  |  |  |
| 16422552 | BGIOSGA034209-TA | 2.09 | 0.001558 |  |  |  |  |
| 16529673 | LOC_Os07g38950.1; BGIOSGA026002-TA | 2.09 | 0.011866 |  |  |  |  |
| 16474499 | LOC_Os03g41170.1; BGIOSGA010260-TA | 2.08 | 0.009427 |  |  |  |  |
| 16510296 | LOC_Os06g01934.1; BGIOSGA022158-TA | 2.07 | 0.001887 |  |  |  |  |
| 16559398 | LOC_Os09g35690.1; LOC_Os09g35690.2; BGIOSGA029409-TA | 2.06 | 0.001932 |  |  |  |  |
| 16416550 | LOC_Os11g03390.1; LOC_Os11g03390.2; BGIOSGA034717-TA | 2.06 | 0.003518 |  |  |  |  |
| 16504362 | LOC_Os05g09740.1; BGIOSGA018662-TA | 2.05 | 0.004423 |  |  |  |  |
| 16425926 | BGIOSGA036949-TA | 2.04 | 0.035384 |  |  |  |  |
| 16476710 | LOC_Os03g53750.2; LOC_Os03g53750.1; BGIOSGA009809-TA | 2.04 | 0.042415 |  |  |  |  |
| 16397467 | LOC_Os01g18240.1; BGIOSGA001872-TA | 2.03 | 0.001412 |  |  |  |  |
| 16501757 | BGIOSGA020320-TA | 2.03 | 0.007549 |  |  |  |  |
| 16415260 | LOC_Os10g38700.1; BGIOSGA031510-TA | 2.01 | 0.013446 |  |  |  |  |
